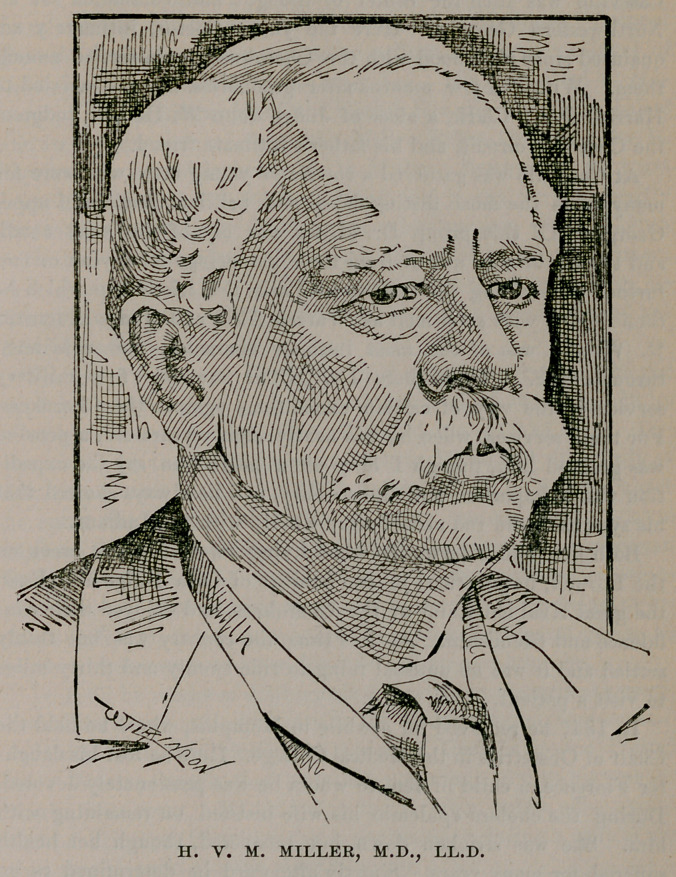# A Sketch of the Life and Character of Dr. H. V. M. Miller

**Published:** 1896-07

**Authors:** Hooper Alexander


ATLANTA

### Medical and Surgical Journal.

Vol. XIII. JULY, 1896. No. 5.

LUTHER B. GRANDY, M.D., MANAGING EDITOR.
M. B. HUTCHINS, M.D., BUSINESS MANAGER.
ALEX. W. STIRLING, M.D., C.M., M.B. (Edin.), D.P.H. (Lond.), MANAGING EDITOR PRO TEM.
COLLABORATORS:
A. W. CALHOUN, M.D., LL.D., VIRGIL O. HARDON, M.D., FLOYD W. McRAE, M.D., AND DUNBAR ROY, M.D.

### ORIGINAL COMMUNICATIONS.

### A SKETCH OF THE LIFE AND CHARACTER OF DR. H. V. M. MILLER.

By HOOPER ALEXANDER.

No account of Dr. Miller's life that is now possible can give any adequate conception of his history. Georgia has no grievance against him except this, that his personal modesty made him rather shrink from than seek that notoriety which most men crave. So far as I know, his only speech ever preserved in print was an eulogy pronounced over the dead body of Alexander H. Stephens. In it he said that Mr. Stephens's highest title to fame was like that claimed by Pericles, who boasted that in a long public life he had done no act that ever caused a citizen of Athens to put on mourning. May I not justly say that the virtue which Dr. Miller ascribed to his great friend was pre-eminently his own?

Unfortunately the details of his life and work will perish with those who knew him. Nay more, because the men who were his active contemporaries are already gone, those details are already largely forgotten. Too unselfish to seek his own aggrandizement, and too modest to give active aid to those who would gladly have preserved some record of his life, to-day the positive side of his history has largely perished in the evanescent memories of mortal men, and the best we now recall of him is that same tribute he accorded the Great Commoner, that he never brought shame or sorrow to any Georgian.

Dr. Miller was born near Walhalla, in South Carolina, April 29, 1814. His father was General Andrew Miller, one of those hardy men, strong in mind and body, who seem to have been so naturally the product of frontier times and conditions; a dominant, self-assertive, self-reliant man of the sort that blaze out the pathways of civilization and lay the foundations for future States; a man of high broad forehead, steel blue eye, and inflexible courage; one of those men who never fail or falter in loyal devotion to a friend. Among the cherished traditions of my own family is a story of how once when he was a member of the General Assembly, and my grandfather, who was his friend, stood in need of his help, he walked from Augusta to Milledgeville to be in time, because the seats in the stage coaches had been anticipated for days. His wife was a most accomplished and cultured lady, a Miss Cheri, of Virginia birth and Huguenot descent. From her Dr. Miller acquired his elegant literary taste and mastery of English. I have often heard him say that her speech was as perfect as Addison's.

Dr. Miller was named in honor of Colonel Homer V. Milton, a distinguished Georgian, and with whom General Miller had served in the second British war. His name therefore did not, as some supposed, mirror any special classical predilection of his parents.

The present territory of Rabun county was acquired by treaty from the Cherokee Indians in 1819. General Miller soon after emigrated from Carolina and settled at the head of that beautiful valley now known as Head of Tennessee, which stretches away in fields of measureless fertility from Rabun Gap northward, shut in by the Blue Ridge on the south, the Cowee on the east, the Nantahala on the west, and blocked by the Smokies on the north, save for one narrow gorge through which the Little Tennessee cuts its way. Amid these inspiring mountain scenes, the subject of this sketch spent his boyhood, and laid the foundations of that superb education which through life made him the oracle sought by many

H. V. M. MILLER, M.D., LL.D.

men. Later his father, again impatient of advancing population, moved further west to the fertile valleys of the present county of Troup, where the son commenced the study of medicine which later he continued at the Medical College of South Carolina, where he graduated with high honors in 1835.

At this time Georgia had just acquired the Cherokee country. Cassville was then the center of thought and commerce for all Northwestern Georgia. Here the young doctor, intimately acquainted since childhood with the Cherokees, again settled among them. Within a few months after graduation he was married to Harriett Perry Clark, a niece of Judge John W. Hooper, judge of the Cherokee circuit, and his father's intimate friend.

At Cassville was gathered a coterie of young men who were for many years the most distinguished and brilliant leaders of upper Georgia. Of this group Dr. Miller was one of the most noted, and though he was absent from their gatherings for a year or two further prosecuting his studies in Paris, the friendship which he then formed with such men as Warren Akin and Judge Augustus R. Wright was of the most intimate character, and continued through life. While here Dr. Miller saw his first military service in the way of certain expeditions against the Cherokees. For these services, when he was nearly eighty years old, a pension was granted him, though I have often heard him say the expedition was unimportant and unnecessary, and he always showed that his sympathies in the matter were wholly with the Indians.

Both here and afterwards when living on Conaseena creek on the Etowah, he practiced in the families of both Ridge and Ross, the great rival chieftains of the Cherokees, and enjoyed their confidence and friendship. At this time the country was but thinly settled and it was no unusual thing to ride twenty and thirty miles to visit a patient.

In 1847 he removed for a while to Memphis, where he held the Chair of Obstetrics in the Medical College. Here he lost his daughter Florence, a child of ten, to whom he was passionately devoted. During the cholera epidemic his wife insisted on remaining with him. She was stricken down, but recovered, though her health suffered for many years. Shortly afterward he determined to go back to Georgia, and this time settled at Rome, then just springing up at the junction of the Etowah and Oostanaula. On a beautiful range of hills about a mile west of Rome he constructed an elegant home, which, in honor of his mother's Huguenot traditions, he called Coligni, and here he remained honored and esteemed by all classes of men until again summoned to arms.

The famous Eighth Georgia Regiment was one of the first at the front and bore the brunt of battle on the left at Manassas. Colonel Francis S. Bartow, its commanding officer, was senior colonel of the brigade and commanded as brigadier. He was killed at Manassas. Three companies of the Eighth Georgia were from Rome and its vicinity, one of them, the Miller Rifles, Captain John R. Towers, was named in honor of Dr. Miller. Bartow had for years been his personal and political friend, and it was, perhaps, partly due to this fact that Dr. Miller went out as surgeon of the Eighth with the rank of major. One of the old members of the regiment said to me to-day, that he never saw as many wounded men in his life as he saw in Dr. Miller's field hospital that bloody day of the first Manassas.

To those who knew Dr. Miller it is needless to say that he did his full duty from the beginning to the end of the war. At its close he was among the highest officers of the medical staff. Impoverished by the result he went back to Rome and cheerfully at fifty began over again. In 1867 he was called to Atlanta where he spent the balance of his life an active lecturer in the Atlanta Medical College of which at his death he had for many years been Dean. Until 1888 he continued in the active practice, occasionally after that date making special visits, one of these being to his life-long friend, the distinguished Dr. Robert Battey, to whom he ministered successfully when his case had seemed hopeless.

In 1868 he rendered distinguished services to Georgia as a member of the Democratic minority in the Constitutional Convention, and afterwards was elected to represent Georgia in the United States Senate, though that body did not admit him to his seat for nearly three years. He held after this no other public office except that of Principal Physician to the penitentiary during a part of the time that General Gordon was Governor.

For thirty years he was a Trustee of the University. Though not a graduate himself of any academic institution, he was intensely concerned on the subject of higher education. To the University he was deeply attached. His last illness found him preparing for his annual visit to Athens and pondering over measures for the good of the school. One of the things which seemed to concern him most was the apprehension that his recovery would be delayed beyond the commencement.

In December, 1893, he had a severe attack of the prevailing influenza called la grippe so severe in fact that for a while his life was despaired of. His devoted wife, with whom he had lived nearly sixty years, in her anxiety and solicitude for his health, overtaxed her strength, was herself stricken down and died. When it became apparent that she must die it fell to my lot to acquaint him with the sad intelligence. It was one of the most distressing duties I ever had to perform. He received the news calmly and with some expression of hopeful doubt. After a little while he rose from his own bed and crossing the hall to her room, lay down beside her, placed his ear to her chest and listened to her breathing. At that time she seemed to be dying, though she lived nearly a week longer. After this examination he went in every day, and until the last continued to express the hope that her splendid constitution would enable her to rally. When she finally died he rose from his sick bed and accompanied her body to Rome where they had always desired to be buried.

Dr. Miller always seemed to me to regard Rome as his home. During his visit there at the burial of his wife he was deeply affected by the troops of friends and of the children and grandchildren of friends who came to visit him. The manifestations of deep-seated affection for him evoked by his bereavement, from a people he had always loved, touched the tenderest chords of his sympathetic nature, and he could rarely ever afterward allude to the visit without a quaver in his voice.

His last illness came on about the first of May in a violent dysentery. His own judgment of his case was evidently correct, though he made no effort to interfere with or make suggestions to the attending physician. To Dr. W. S. Kendrick, who treated him and to whom he was deeply attached, he said one day, "Doctor, I think your treatment of my case is correct. If the measures you are trying do not relieve me nothing else will." He studiously refrained from counting his own pulse or inquiry about it. During the worst of his attack he refrained almost entirely from food. His own simple habits and Dr. Kendrick's skill finally seemed about to win the battle. On the 29th he seemed to have entirely recovered from the dysentery and got up and dressed. On the 30th and 31st he was up and about the house. In the afternoon he complained of fatigue and at the suggestion of a friend lay down on his bed. As his head touched the pillow the splendid but worn machinery of his body just stopped and he was dead.

Dr. Miller is said to have been a great orator. I never heard him speak but two or three times. In these he justified the reputation he bore. In 1886 he made several speeches in behalf of one whom he had loved for years. In one of these he told an anecdote that turned the tide of a gubernatorial election. The whole State shook with laughter over the story, and it silenced an adverse accusation. His speeches were never prepared. Few were reported, and such as were were not printed.

He was the wisest man I ever knew. His judgment of men was keen, his foresight of events marvelous. His education was self-acquired, his learning prodigious, his memory astounding.

In medicine he was pre-eminently successful, but believed little in drugs. I have heard him say it was doubtful if medicines had not done about as much harm as good. Of medical theories and hypotheses I am, of course, uninformed, but I observed that some of the opinions now almost universally adopted by the profession, he regarded with a sort of good-humored incredulity and amusement, though I do not remember to have heard him say more in doubt or question than this: "Well, the theory is not proven yet." When some French physician announced the discovery of the elixir of youth he said: "You will presently see a lot of doctors poisoning people by hypodermic injections in order to get their names in a newspaper." When the merit of some remedy was argued, about which he was skeptical, and cases were cited where it had wrought cures, he would say: "The Hottentots have proven by experiment that a loud noise will remove an eclipse from the sun."

In opinion he was broadly tolerant, possessed of the simplest faith in God, and more than any man I ever saw, obeyed the injunction to love the Lord with all his strength and his neighbor as himself. In church membership he was a Methodist, and adhered closely to his church organization, though he always claimed that the present form of Methodist church government by bishops was unscriptural and opposed to Wesley's teaching. It was a favorite theme with him also to tease his brethren of the Methodist pulpit by quoting an entry from Wesley's journal about having baptized somebody in Savannah "by immersion according to the Word of God and the practice of the early Christians." It was another of his favorite themes to insist that the Presbyterian Shorter Catechism was the only proper religious system on which to bring up the young. From all which things I am lead to conclude that he believed the Word of God a bigger and a broader thing than any church.

His private charities were broad and generous, and he never allowed his right hand to know what his left hand did. All his life he overtaxed his own resources in the sympathetic helpfulness that made it impossible for him to refrain from giving help where he saw that help was needed.

In personal character Dr. Miller was superb. Brave as Julius Cæsar, he was as gentle as any woman; strong in his own purposes and conviction, he was patient and long-suffering with the errors of his fellow-man, and never lost hope of their ultimately getting right; generous, tender, faithful and kind, with no vestige in his nature of anything mean or little or low, his rule of life was to do to others as he would have them do to him, and this was why he was always recognized as being so completely and essentially a gentleman. The one thing in this world that he hated was a lie; the one thing that he scorned with all the splendid scorn of his big, clean soul was the man who would tell a lie. Upon that which was low and false, and upon men who were low and false, he looked with unutterable loathing.

The world is in some measure better for his having lived in it, and none who ever knew him have ever had cause to reget it. He was given to live years beyond the allotted time of man. Those years he gave to good; not ostentatiously, but humbly and quietly, long expecting and listening for his Master's call, ever ready for it but never impatient. Like one of his favorite characters in literature, he just waited for the roll-call, ready to answer when his name was sounded, "adsum." A modest citizen, a Christian hero, a loyal gentleman has quietly ended his appointed days.

## Figures and Tables

**Figure f1:**